# The Prognostic Value of Global DNA Hypomethylation in Cancer: A Meta-Analysis

**DOI:** 10.1371/journal.pone.0106290

**Published:** 2014-09-03

**Authors:** Jinhui Li, Qingyuan Huang, Fangfang Zeng, Wenxue Li, Zhini He, Wen Chen, Wei Zhu, Bo Zhang

**Affiliations:** 1 Department of Preventive Medicine, School of Public Health, Sun Yat-sen University, Guangzhou, P.R. China; 2 Department of Toxicology, Guangzhou Center for Disease Control and Prevention, Guangzhou, P.R. China; 3 Department of Thoracic Oncology, State Key Laboratory of Oncology in South China, Sun Yat-sen University Cancer Center, Guangzhou, P.R. China; 4 Department of Epidemiology, The First Affiliated Hospital, Sun Yat-sen University, Guangzhou, P.R. China; 5 Guangdong Provincial Key Laboratory of Food, Nutrition and Health, Department of Medical Statistics & Epidemiology, School of Public Health, Sun Yat-sen University, Guangzhou, P.R. China; University of Nebraska Medical Center, United States of America

## Abstract

**Background:**

Aberrant methylation of the global genome has been investigated as a prognostic indicator in various cancers, but the results are controversial and ambiguous.

**Methods and Findings:**

This meta-analysis presents pooled estimates of the evidence to elucidate this issue. We searched the electronic databases: PubMed, Embase, ISI Web of Science and the Cochrane library (up to August 2013) to identify all of the relevant studies. The association between the level of surrogates' indexes of genome-wide hypomethylation (LINE-1, Alu and Sat–α) and the overall survival (OS) of cancer patients was examined. In addition, the pooled hazard ratios (HRs) with their 95% confidence interval (95%CI) were calculated to estimate the influences through fixed-effects and random-effects model. Finally, twenty studies with total population of 5447 met the inclusion criteria. The results indicate that the summary HRs for the studies employing LINE-1, Alu, and Sat-α repetitive elements also show that the global DNA hypomethylation have significant desirable effects on the tumour prognostic value. The pooled HRs (and CIs) of LINE-1, Alu and Sat-α were 1.83 (1.38–2.44), 2.00 (1.16–3.45), and 2.92 (1.04–8.25), with a heterogeneity measure index of I^2^ (and *p*-value) shows of 66.6% (*p* = 0.001), 57.1% (*p* = 0.053) and 68.2% (*p* = 0.076) respectively. The meta-regression and subgroup analysis indicated that the percentage of hypomethylated sample of cancer patients is one source of heterogeneity.

**Conclusion:**

Our meta-analysis findings support the hypothesis that the global DNA hypomethylation is associated with a detrimental prognosis in tumour patients.

## Introduction

Cancer is a leading cause of death worldwide, accounting for 7.6 million deaths (approximately 13% of all deaths) in 2008, and this number is projected to continue increasing to an estimated 13.1 million deaths in 2030 [1]. The reliable identification of molecular prognostic biomarkers is significant for the facilitation of the rational choice of potential therapeutic methods in cancer treatment and for improving the prognosis of cancer patients [2]–[4].

DNA methylation is an epigenetic modification involving a covalent addition of a methyl group (CH_3_) from the methyl donor S-adenosylmethionine (SAM) to the 5-position carbon of the pyrimidine ring of a cytosine base and formation of 5-methylcytosine (5meC), typically occurring in CpG dinucleotide contexts in mammal cells. Although CpGs are disproportionately concentrated in enriched regions referred to as CpG islands (CGIs), which tend to be differentially located in the promoter regions of genes, 70–90% of all CpGs in the human genome are in regions of large repetitive sequences (i.e. centromeres and retrotransposon elements) and are typically methylated under normal conditions [5]. Then the methylation level of the repetitive elements including long interspersed element-1 (LINE-1), Alu element (Alu), and Satellite-α (Sat-α), which constitute 11%, 17%, and 4% of the genome respectively, and cover more than 30% of the total CpG sites in the genome, may serve as surrogate indexes as genome-wide DNA methylation level [6].

With the recent developments in the high-throughput and high-resolution methods for DNA methylation analysis such as pyrosequencing and luminometric methylation assays (LUMAs), global DNA methylation level was fully analysed in various tumours. More and more researchers found that DNA hypomethylation is inversely associated with tumour progress through promoting carcinogenesis by increasing DNA recombination or by direct and indirect effects on gene expression, such as overexpression of some imprinted genes, activation of genes associated with tumour invasion or metastasis and so on [7].

Although numerous studies have attempted to assess the prognostic value of DNA hypomethylation in diverse cancers, identification of the results remain controversial and ambiguous as following: several studies have found that global DNA hypomethylation was a prognostic factor for dismal outcome, other studies, however, drew beneficial conclusions [8]–[10] or found no significant association [8], [9], [11]–[14]. To clarify this question, we conducted this meta-analysis to appraise the prognostic value of global genomic hypomethylation in various tumours.

## Materials and Methods

### Search strategy and selection criteria

We followed the guideline of MOOSE (Meta-analysis of Observational Studies in Epidemiology) [15] and PRISMA (Preferred Reporting Items for Systematic Reviews and Meta-analysis) [16] to systematically conduct and report this meta-analysis. Two reviewers (Jinhui Li and Qingyuan Huang) independently performed a systematic literature search of the following electronic databases to identify studies that assessed the prognostic utility of global DNA (including its surrogate markers: Alu, LINE-1 and Sat-α) hypomethylation in patients with any type of carcinomas without language limitation: PubMed, Embase, ISI Web of Science and the Cochrane library (last search was on August 2013).

Based on the Medical Subject Headings (MeSH) and corresponding search terms, our search strategy was the following: (whole genome “DNA” OR “LINE-1” OR “Sat-α” OR “Alu” AND “hypomethylation*”) AND (“Prognosis” [Mesh] OR “Survival Rate” [Mesh] OR “Mortality”[Mesh]) AND (“tumour” OR “cancer”). Over the same period, we also manually searched for abstracts from selected bibliographies carefully to identify additional studies. Only those papers restricted to human studies and published as full-text articles in English or Chinese were included as primary candidates. In addition, the identified studies were not limited by the year of publication.

The initial set of eligibility criteria applied was the following: (1) The articles represented original epidemiological studies that assessed the level of global DNA hypomethylation and its corresponding cut-off standard, including its surrogate markers (LINE-1, Alu, and Sat-α); (2) The outcome of the overall survival (OS) used in the studies could be accepted; (3) The studies reported a hazard ratio(HR) and the corresponding 95% confidence interval (95%CI) directly or provided the relevant data or survival curve, which we can use to estimate the HR and 95% CI; (4) The study sample size was higher than thirty individuals.

### Data extraction and management

We designed a standard form with the Access Database of the Microsoft Office Suite that facilitated the extraction and input of the data into a corresponding blank plot. The database was used to record the most relevant data, which encompassed (1) the general information of the studies (publication year, mean age and follow-up, number of patients, detected materials and methods, treatment administered, cancer types, and evaluation score) and (2) the HRs and corresponding 95% CIs for the OS of patients with a global hypomethylation level compared with patients without global hypomethylation level. Throughout the procedure, one review author (Jinhui Li) mined the data from the included studies and another author (Qingyuan Huang) checked the extracted data. All minor disparities were resolved by discussions between the authors.

Studies providing univariate or multivariate analysis (or both) results for the survival were used to aggregate the survival data. When related data were unavailable directly from the studies, we calculated the corresponding HR and 95%CI using the indirect methods described by Tierney *et al* [17]. This method allows the use of the parameters given in the publications, such as the O-E statistic and variance, the number of patients who exhibited beneficial or harmful prognosis and the log-rank statistic or its *p*-value to estimate the HR and its variance using the established formulas. If the manuscripts only offered the survival curve, we used the Engauge 4.1 software to obtain the individual survival data at certain specified times, and then the Excel tables provided by Tierney *et al* were used to obtain the summary HRs and CIs.

### Quality Score Assessment

To evaluate the methodological quality of the studies, two authors (Jinhui Li and Qingyuan Huang) scored them according to the REMARK guidelines. The REMARK guidelines include the following four parts with multiple sub-items: introduction, materials and methods, results, and discussion. Each of these parts contains a few items, such as markers examined, biological materials used and standard prognostic variables among others. The two investigators scored the quality of each publication independently, and then reached a consensus value for each item, using a checklist in which one point was allocated to each reported one. A higher score represented a better methodological quality.

### Statistical analysis

To quantitatively combine the survival data, we extracted the HRs and their 95% CIs to assess the impact of the global hypomethylation status on tumour prognosis. I^2^ was adopted to assess the heterogeneity among the studies [18]. I^2^ values of 25%, 50%, and 75% correspond to the cut-off points for low, moderate, and high degrees of heterogeneity [19]. If the assumption of homogeneity was supported (I^2^>25%) , the random-effects model was adopted to calculate the HR according to the DerSimonian-Laird method [20]. Otherwise, the fixed-effects model (Mantel–Haenszel method) was used directly [21]. In addition, we also explored the heterogeneity through single variable meta-regression analysis and subgroup analyses using the factors mentioned above. Funnel plots with Egger's regression were adopted to examine the effect of publication bias. To determine whether the conclusions could be affected by removing one or two studies, we conducted sensitivity analyses. All differences with a *p-*value that was less than 0.05 were considered as significant. All of the mentioned above were conducted using the STATA 12.0 software platform (Stata Corporation, College Station, TX, USA).

## Results

### Search results and characteristics

**Figure 1:**
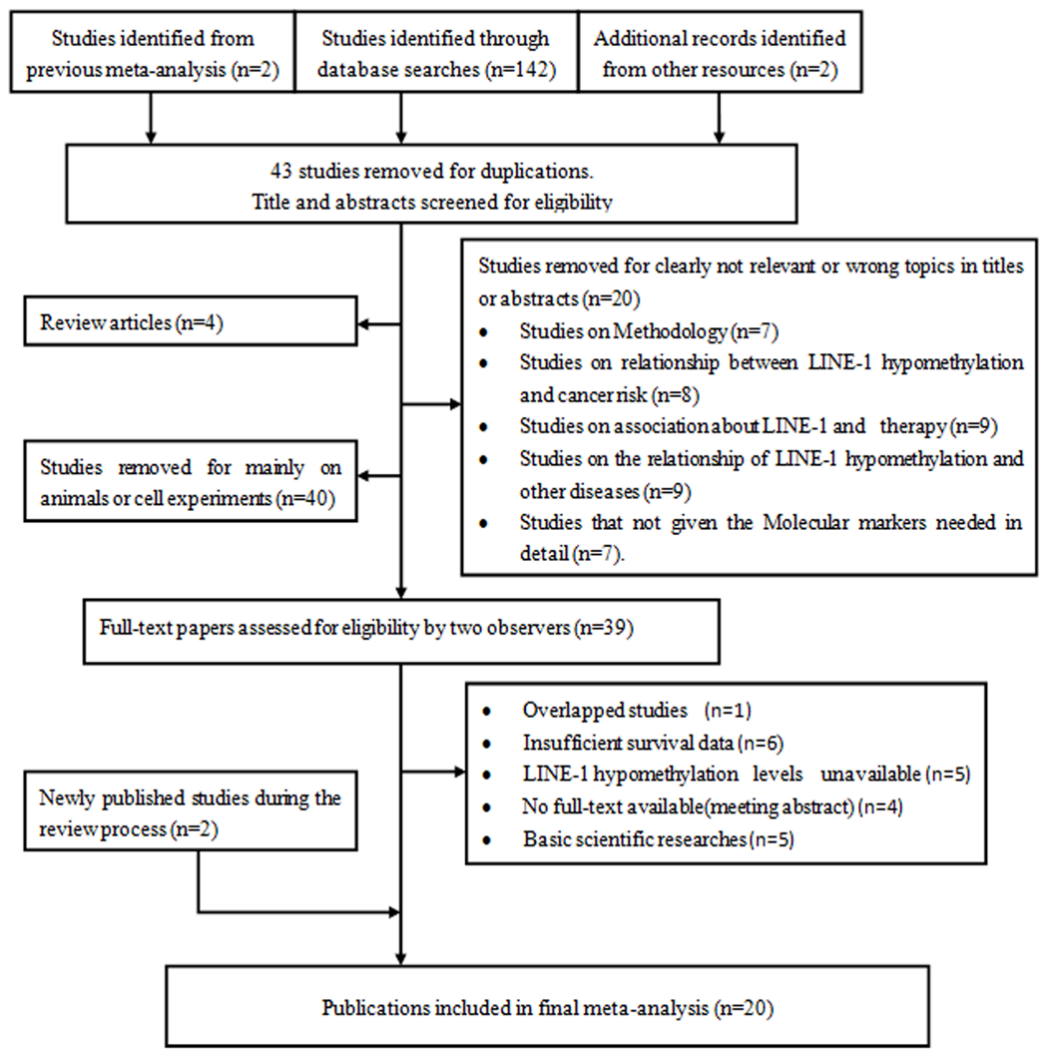
Flow diagram illustrating the screening and selection process.

A total of 146 relevant citations were identified for initial review using the search strategies described previously. Based on a review of the titles and abstracts, 107 of the original studies were removed because they did not meet the initial eligibility criteria. The full text of the remaining 39 original studies was then reviewed, and 14 of these articles were eliminated on the basis of inadequate data, and one article was excluded because it was an overlapping publication. Six studies did not include sufficient survival data, five studies did not contain detailed information on global methylation, four studies were just seminar abstracts with no method to obtain the full texts, and the remaining five studies were basic scientific research studies. Thus, after the application of the inclusion and exclusion criteria given above, 20 studies were included in the analysis (Figure 1) [8]–[14], [22]–[33].

The main features extracted from the 20 studies that were included in the meta-analysis are summarised in Table 1. Among these studies, 15 trials were on LINE-1, and three studies focused on LINE-1 and Alu. One study was about Alu and Sat-α, and another one study examined the three indexes. The total study population was 5447, with a mean of 183 subjects per study (range, 42 to 2068). The mean age of the subjects was 58.6 years, with a range of 42 to 72 years old. The trials were conducted in six countries (Korea, USA, China, Thailand, Japan and Italy), and the publication year ranged from 2008 and 2013. The global DNA methylation level was detected through four main methods (pyrosequencing methylation, BSP, MSP and COBRA) with two types of hypomethylation dividing level. The analysis of the biological materials revealed that 15 studies used tissue samples, two used blood samples, one performed cell culture of tissues samples, and another one used bone marrow samples. The mean evaluation score was 16.5 with a range of 14 to 20. And the tumors contained various types, such as tumors of the digestive system, respiratory system tumors and other tumors. In addition, we were able to directly obtain the HRs and CIs from 16 of the studies, whereas we had to extrapolate the HRs and CIs of the other four trials from the graphical expression of the survival distributions.

**Table 1:** Major features of the included studies.

Study	Location	Number	Age (mean)	Cancer Type	Follow-up (months)	Materials	Detection Method	Measurement	Hypomethylation Cut-off	Rate (%)	Quality Score
JM Bae 2011	Korea	198	59	GC	46(1–72)	tumor tissue	Pyrosequencing	LINE-1	dichotomy	55.79	17
								Alu	dichotomy	17.37	
ZZ Zhu 2011	USA	722	72	all cancers	85(2–118)	blood	Pyrosequencing	LINE-1	dichotomy	68.1	17
								Alu	dichotomy	26.0	
F Ohka 2011	Japan	54	59	GBM	172.8(8.9–336.8)	tumor tissue	Pyrosequencing	LINE-1	dichotomy	63.5	15
F Ohka 2011	Japan	57	42	LGG	172.8(8.9–336.8)	tumor tissue	Pyrosequencing	LINE-1	dichotomy	68.9	15
H Shigaki 2012	Japan	203	70	GC	34.8(13.6–56)	tumor tissue	Pyrosequencing	LINE-1	dichotomy	72.3	17
Y Aoki 2012	Japan	81	67.5	MM	20(3–40)	bone marrow	BSP	LINE-1	dichotomy	41.7	14
								Alu	dichotomy	37.1	
								Sat-alphaα	dichotomy	39.8	
S Hoshimoto 2012	USA	126	63.4	Melanoma	70(3–140)	tumor tissue	MSP	LINE-1	dichotomy	32	15
L Sigalotti 2011	Italy	42	54	Melanoma	15.3(11.0–31.5)	tissue-cell culture	Pyrosequencing	LINE-1	dichotomy	31.13	16
K Saito 2010	Japan	379	48	NSCLC	45.5(2-149)	tumor tissue	BSP	LINE-1	dichotomy	81	18
S Fabris 2011	Italy	81	65	CLK	24(12-120)	blood	Pyrosequencing	Alu	dichotomy	21.4	15
								Sat-alphaα	dichotomy	84.0	
K Nosho 2009	USA	2068	65.6	CRC	144(24–312)	tumor tissue	Pyrosequencing	LINE-1	dichotomy	61.50	18
CS Zhang 2011	China	95	49.86	HCC	25(0–50)	tumor tissue	Q-MSP	LINE-1	dichotomy	75.5	16
YY Rhee 2012	Korea	207	55	CRC	46(1–76)	tumor tissue	Pyrosequencing	LINE-1	dichotomy	53	20
								Alu	dichotomy	18.60	
J PATTAMADILOK 2008	Thailand	59	50	OC	59(0–108)	tumor tissue	COBRA	LINE-1	dichotomy	34.87	17
A Murata 2013	Japan	74	65	CRC	46.8(50.4–144)	tumor tissue	Pyrosequencing	LINE-1	dichotomy	52.80	18
S Iwagami 2013	Japan	217	65.9	ESCC	31.2(3–66)	tumor tissue	Pyrosequencing	LINE-1	quatile	65.10	19
CS Furniss 2008	USA	193	59	HNSCC	36(0–60)	tumor tissue	COBRA	LINE-1	dichotomy	45	15
Z Xv 2010	China	105	50	HCC	20(0–40)	tumor tissue	MSP	LINE-1	dichotomy	74.65	16
Annek Q 2012	USA	243	55	BC	7.5(0–15)	tumor tissue	AQAMA	LINE-1	quatile	18.5	16
S Ogino2008	USA	243	NA	CRC	60(0–120)	tumor tissue	Pyrosequencing	LINE-1	quatile	45	17

Abbreviations: MSP = Methylation-specific PCR; Q-MSP = Quantitive MSP; BSP = bisulfite sequencing PCR; COBRA = Combined bisulfite restriction analysis ; GC = Gastric Cancer;; GBMs = Glioblastoma Multiformes; LGG = Glioblastoma Glioblastoma multiformes; MM = Multiple Myeloma; NSCLC = Non-Small Cell Lung Cancer; CRC = Colorectal cancer; HCC = Hepatocellular Carcinoma; HNSCC = Head and Neck Squamous Cell Carcinomas; CLL = chronic lymphocytic leukemia; ESCC = Esophageal Squamous Cell Carcinoma; OC = Ovarian Cancer; BC = Breast Cancer; CLK = chronic lymphocytic leukemia.

### Meta-analysis

**Figure 2:**
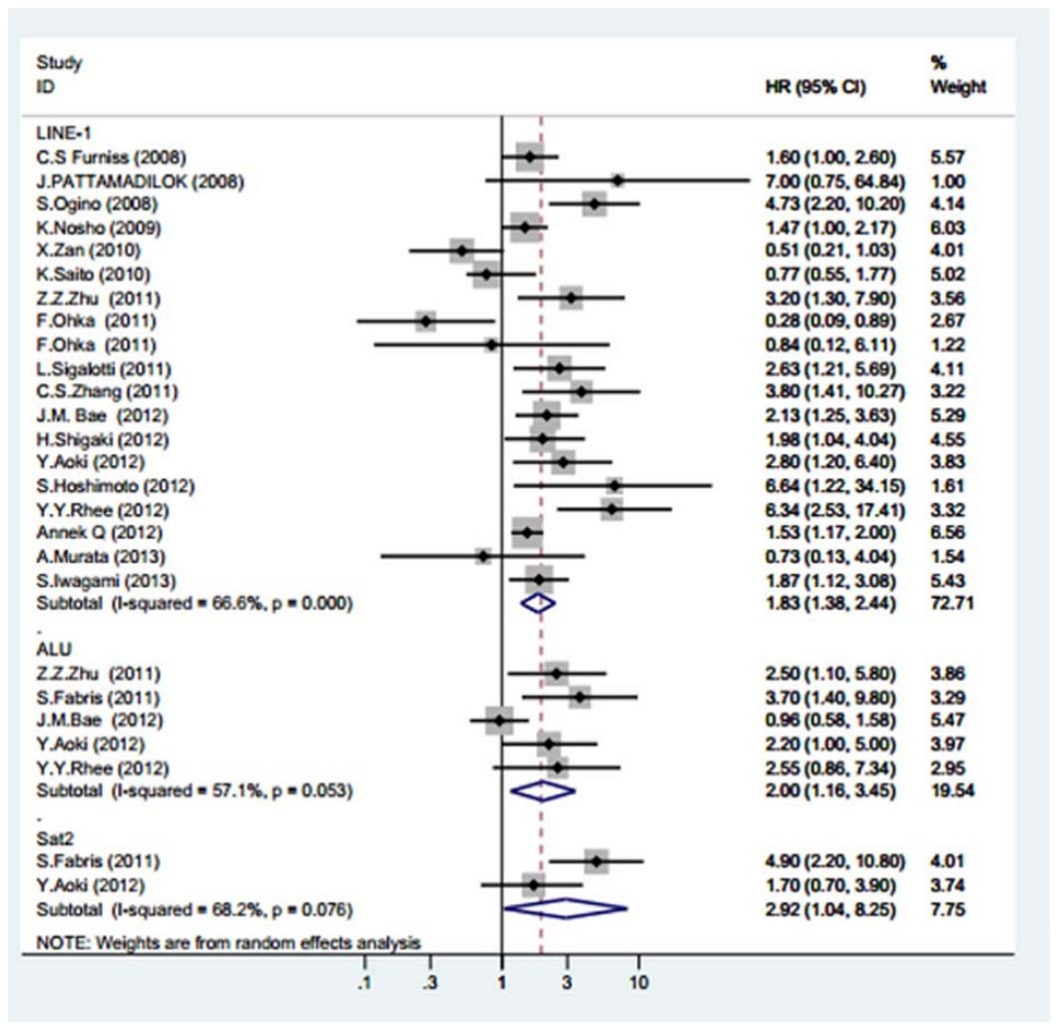
Meta-analysis of the forest plot showing the association between the genome-wide DNA hypomethylation and the overall cancer survival. The squares represent the size of the study and are centred on the HR, and the whiskers represent the 95% CIs. A random effects (RE) model was used.

A meta-analysis was used to analyse the studies that adopted the surrogate indexes (LINE-1, Alu and Sat-α) for the determination of the degree of genomic methylation. Using the random-effects model due to the significant heterogeneity of the studies, dismal survival outcomes were observed for tumour patients with global DNA hypomethylation. The summary HRs obtained in the studies employing LINE-1, Alu and Sat-α repetitive elements also indicated that global DNA hypomethylation has significant negative effects on tumour prognosis. The pooled HRs and CIs were 1.83 (1.38–2.44), 2.00 (1.16–3.45), and 2.92 (1.04–8.25) with I^2^ values of 66.6%, 57.1%, and 68.2% respectively. Figure 2 shows the results of the forest plot explained above.

Due to the relatively high heterogeneity exhibited in the trials aggregated with respect to the overall survival, meta-regression and subgroup analyses were conducted to explore the heterogeneity of the covariates including the study location , number of patients, mean age, follow-up, biological materials, hypomethylation rate and cut-off, methylation detection method, cancer types, treatment information and quality score. Because of the limited number of studies that used Alu (n = 5) and Sat-α (n = 2) for the analysis of global hypomethylation and the OS relationship, we only performed meta-regression for the publications on LINE-1 as the surrogate of global genomic methylation. Ultimately, the methylation level was found to be a source of heterogeneity (*p* = 0.067), whereas the others variables were not (Table 2). The subgroup analyses of the overall survival and global DNA hypomethylation status detected a little significant correlation in the patients with a global DNA methylation rate less than 50% (HR, 2.52; 95%CI, 1.72–3.71; I^2^, 54.2%).However, after stratification by mean age, year of publication, mean follow-up, biological samples used, hypomethylation cut-off, and test method, no significant results were found.

**Table 2:** Meta-regression and subgroup analysis of the studies reporting the association of LINE-1 hypomethylation and overall survival of cancer patients.

*Stratified study*	*No. of studies*	*No. of patients*	*Fixed-Model (HR, 95%CI)*	*Random-Model (HR, 95%CI)*	*Meta-regression p value (%)*	*HG I^2^ (%)*	*HG p-value*
**Year**					0.362		
<2011	7	1342	1.40(1.10,1.77)	1.46(0.83,2.58)		77.5	0.001
> = 2011	12	2247	1.88(1.57,2.24)	2.09(1.51,2.91)		56.9	0.006
**Hypomethylation cut-off**					0.603		
Dichotomy	16	2886	1.65(1.37,1.98)	1.76(1.22,2.52)		67.5	0.001
Quartile	3	703	1.76(1.40,2.20)	2.15(1.25,3.71)		73.3	0.001
**Hypomethylation rate**					0.067		
< = 50%	7	1010	1.92(1.57,2.35)	2.52(1.72,3.71)		54.2	0.033
>50%	12	2579	1.48(1.21,1.82)	1.40(0.92,2.14)		71.8	0.001
**Study race**					0.300		
Yellow	12	1729	1.76(1.47,2.11)	2.08(1.50,2.89)		55.8	0.035
White	7	1860	1.59(1.26,2.00)	1.57(0.97,2.53)		72.4	0.001
**Age**					0.423		
< = 60	9	123	1.52(1.27,1.82)	0.33(0.10,0.76)		73.7	0.001
>60	9	3438	1.86(1.45,2.72)	1.93(1.44,2.58)		15.9	0.308
**Method**					0.759		
Pyrosequencing	12	2365	1.90(1.57,2.30)	0.54(0.15,0.93)		60.8	0.003
Others	7	1224	1.45.(1.17.,1.80)	1.70(0.96,3.02)		73.2	0.001
**Bio-samples**					0.304		
Tissue	16	2617	1.61(1.38,1.87)	1.55(1.11,0.93)		69.3	0.001
Blood	3	972	2.84(1.76,4.59)	2.84(1.76,4.59)		0.00	0.948
**REMARK Score**					0.728		
<17	10	1191	1.73(1.24,2.42)	1.66(1.16,1.88)		66.6	0.01
> = 17	9	2398	1.68(1.44,1.97)	1.89(1.36,2.62)		69.1	0.001
**No. of patients**					0.536		
>200	9	1031	1.68(1.31,2.17)	1.65(1.00,2.70)		66.1	0.001
<200	10	2558	1.69(1.43,2.01)	1.99(1.38,2.87)		71.3	0.001
**Types of cancers**					0.972		
digestive system,	8	2873	1.89(1.50,2.39)	2.07(1.24,3.44)		74	0.001
respiratory system tumors	3	789	1.40(1.04,1.88)	1.35(0.82,2.23)		64.1	0.061
other tumors	8	1384	1.70(1.36,2.13)	1.95(1.13,3.39)		63.1	0.008

Abbreviations: CI = confidence interval; HR = hazard ration; HG: heterogeneity.

### Publication bias and sensitivity analysis

**Figure 3:**
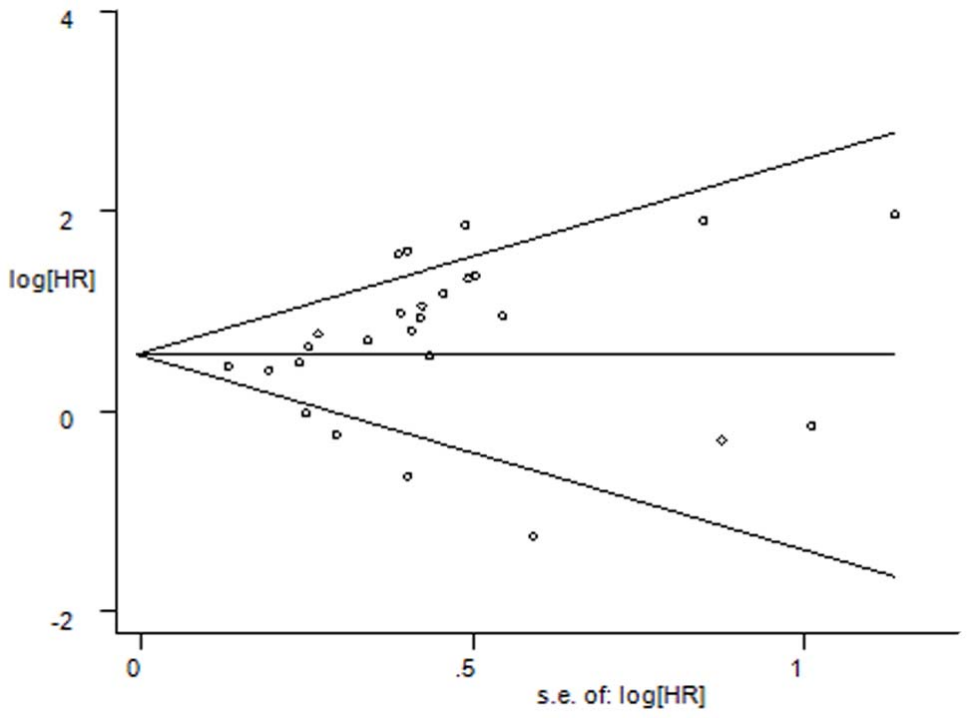
Funnel plot for the visual assessment of the presence of publication bias associated with all of the studies included in the meta-analysis. The funnel graph plots the log of the hazard ratio (HR) against the standard error of the log of the HR (an indicator of the sample size). The open circles indicate the individual studies. The line in the centre represents the pooled HR. Egger's test for publication bias has no significance (p = 0.322).

We chose Egger's test to evaluate the publication bias. The funnel plots revealed that all of the studies included revealed no evidence of obvious asymmetry (*p* = 0.142; Figure 3). Moreover, the sensitivity analysis revealed that the omission of any individual study would not affect the overall results.

## Discussion

To the best of our knowledge, this meta-analysis is the first study to systematically assess the association between global DNA methylation level and tumour prognosis. Our study aggregated the outcome of 5447 samples from 20 individual studies. In summary, all three surrogate indexes, LINE-1, Alu and Sat-α had indicative effect on tumour prognosis, and are potentially independent prognostic biomarkers [22], [25].

The initiation mechanism of global DNA hypomethylation remains poorly understood and several factors have been reported to be implicated in such processes [28]. Environmental factors [34], [35], chemical toxicants [36]–[38], diet and nutrition [39] can perturb the underlying mechanism of genome methylation and can alter the level of global DNA methylation [40]. Enzymes related to DNA methylation, such as DNMT1, DNMT3a, DNMT3b [41], and TET1–3 [42], [43] may be the targets of exogenous or endogenous compounds and the disturbance of them can result in DNA hypomethylation.

The mechanism by which global DNA hypomethylation confers a poor tumour prognosis remains to be fully explored. Genome-wide DNA hypomethylation has been showed to be associated with genomic instability. The global loss of methylcytosine has been proposed to compromise gene repression in genomic regions that are usually silent in normal cells, and this effect may result in the re-expression of proto-oncogenes or imprinted genes, as well as the activation of viral and parasitic transposons, all of which would contribute to genomic instability [29]. Besides, DNA hypomethylation has been consistently demonstrated to increase the immunogenicity and immune recognition of cancer cells through the up-regulation of different molecules involved in antigen pro-ceding and presentation, including HLA class I antigens and co-stimulatory molecules [44]. Another explanation is that global hypomethylation may affect transcriptional dysregulation, then proto-oncogenes endogenous retroviruses, and it is also possible that transposable elements may be actived, and these might affect the tumour's aggressiveness [25].

In this analysis, heterogeneity persisted in the subgroup of the index LINE-1 with an I^2^ of 66.6%. The source of inter-study heterogeneity present in the OS found in this analysis was assessed through meta-regression and subgroup analysis. The results indicate that the percentage of hypomethylated sample of cancer patients may account for part of the inter-study heterogeneity (*p* = 0.067). Moreover, the subgroup analysis indicates that the percentage of hypomethylated sample of cancer patients had an obvious relationship with the patients' prognosis in the group with a hypomethylation level less than 50% (HR, 2.52; 95% CI, 1.72–3.71) and not in the group with a hypomethylation level higher than 50%. Therefore, random models were adopted. This different may be attributed to several reasons, one of which is the lack of detail information of medicine those patients used. As methylation is reversible, if patients had used drugs that affect DNA methylation, the epigenetic biomarker will be affected by treatment. In addition, the biological samples used may affect the methylation rate. Besides, demethylation may also be cancer-specific, i.e., some genome sequences may exhibit differential susceptibility to DNA hypomethylation. Moreover, the degree of global hypomethylation may markedly depend on the cancer histological subtype [29]. Totally, the pooled analysis of all of the studies on different types of cancers used in this meta-analysis demonstrates the predictive value of global DNA hypomethylation for detrimental survival in cancer patients. However, even though some factors had no significant influence, we cannot completely exclude the possibility that some of these covariates may potentially account for part of the heterogeneity, as the power of meta-regression analysis was known to be low. In addition, due to the limited number of studies on Alu and Sat-α included, meta-regression and subgroup analyses could not be conducted for these indexes based on stability of the outcome. Moreover, the subgroup analysis of the methods used to assess DNA methylation status revealed that the pyrosequencing method was significantly better (HR, 0.54; 95% CI, 0.15–0.93) for the examination of the methylation status of biosamples compared with other methods. As for the cut-off value of hypomethylation, the median was used in the vast majority of the included studies, and subgroup analysis of “dichotomy” also showed meaningful predictive significance of poor prognosis (HR: 2.15; 95%CI: 1.25–3.71). Thus, pyrosequencing with median as a cut-off criterion of hypomethylation might be the optimal choice for clinicians who need to predict patient prognoses based on their genome methylation statuses. This finding is in agreement of previous reports those showed that pyrosequencing is an ideal tool for the discrimination of the methylation status of patients with different clinical characteristics [45], [46].

As all of the three indexes exhibited the corresponding predictive value, and in various clinical cohort and case-control studies, the LINE-1 has been adopted as a predictive biomarker for the survival of tumour patients [45], [47], we might concluded that LINE-1 alone or accompanied by other two biomarkers should be the surgeons' appreciation. These indexes are common in most cancer types [48], and some publications indicate that the prediction of the effect will be better by combining detection of two or three indicators [11], [22], [49], although these still need further studies. Besides, some clinical trials about methylation are under researches, such as epigenetic drugs (e.g., DHA, IHD or other clinical trials et al), combined with these predictive biomarkers, will shed light on these malignant tumour patients and clinical doctors [50], [51].

The sensitivity analysis performed in this study revealed that our pooled estimate of the effect was robust and did not change appreciably in the various scenarios tested. Moreover, the meta-analysis included data from a wide range of countries, which indicates that the findings are more representative. This may be attributed to the fact that we followed these two guidelines rigorously and used a strict search strategy and selection criteria. Moreover, the most important step of the search strategy was that we did not limit the language to English. This resulted in the evaluation scores being relatively higher, i.e., the qualities of the studies selected were greater. However, the inclusion of unpublished studies and conference abstracts in our analysis, assuming that the corresponding methodology assessment and meta-analysis can be performed, would improve the results.

We acknowledge that our meta-analysis suffered from several limitations despite our attempts to perform a comprehensive analysis. One of the key limitations is the heterogeneity of the three index groups. The LINE-1, Sat-α, and Alu repetitive elements exhibited median heterogeneity (I^2^ values of 66.6%, 57.1% and 68.2%, respectively). This may due to the fact that the meta-analysis was a study analysis and not an individual analysis. Besides, the sample size of indexes of Sat-α, and Alu are a little small (5 and 2 respectively) to be particularly powerful to explore the sources of their heterogeneities may existed even though the quality of these papers is great. However, our comprehensive analysis revealed that the hypomethylation rate is a source of heterogeneity and we provided general explanations for this discovery. Moreover, only one of the studies included in our meta-analyses used method of Q-MSP, and its removal did not affect the results, as determined through the sensitivity analysis was unaffected by removal of it. Finally, although Egger's test suggested a *p-*value of  = 0.142, the funnel plot provides some slight evidence of asymmetry between the included studies, which indicates that some epidemiological research bias exists. Some of the limited sample sizes may inevitably increase the bias risk or the risk of random errors because only 20 studies (19 publications) were included in the meta-analysis. However, because we performed an extensive examination of the literature with strict evaluation criteria, the scores of these studies were relatively higher. The mean score was 16.5 (range of 14 to 20) according to the REMARK guidelines. Therefore, all of the studies were of good quality.

Based on the results of this meta-analysis, we support the hypothesis that tumour patients with global hypomethylation in all of the populations considered have dismal prognoses. From the point of view of clinicians, the use of “median” as hypomethylation benchmark, and using pyrosequencing to detect the LINE-1 alone or combined with other two biomarkers methylation level of biological samples from a patient may be a useful way to forecast their patients' outcome, and if low methylation is detected, a strengthening treatment, such as the use of postoperative radiotherapy and chemotherapy [52], [53], or some type of intervention treatment and immune-therapy [7], [54] should be considered. However, further validation is required to assess whether this epigenetic biomarker should be used in routine clinical application as a prognostic tool for patients with various tumours. Future studies should have a stricter design, larger sample sizes (to increase the statistical power of the results), a uniform manner of analysing the survival outcomes and the level of hypomethylation, and concordant bio-samples. In addition, future studies require the use of a long and specified follow-up period to confirm these results.

## Supporting Information

Checklist S1 **PRISMA Checklist.**
(DOCX)
